# Reviewed article: Nery DP, Andrade ACS, Rocha DS, Barbosa MCR, Gomes RS, Bezerra VM. Trend in neonatal mortality and preventable neonatal deaths: a time series analysis, Bahia, 2010-2020. Epidemiol Serv Saude.2025:34;e20240651

**DOI:** 10.1590/S2237-96222025v34e20240651.a

**Published:** 2025-09-29

**Authors:** Eunice Francisca Martins

**Affiliations:** 1 Universidade Federal de Minas Gerais, Belo Horizonte, MG, Brazil

## Peer review A

## Questionnaire

Does the manuscript contain new and significant information to justify publication? No

Does the Abstract (Summary) clearly and accurately describe the content of the article? Yes

Is the problem significant and concisely stated? No

Are the methods described comprehensively? Yes

Are the interpretations and conclusions justified by the results? Yes

Is adequate reference made to other work in the field? Yes

## Manuscript Structure

Length of article is: Adequate

Number of tables is: Adequate

Number of figures is: Adequate

Please state any conflict(s) of interest that you have in relation to the review of this paper. None

Interest: Good

Quality: Good

Originality: Below Average

Overall: Average

Recommendation: Major Revision

## Comments

Caro(a)s autore(a)s,

A definição do problema de pesquisa pode ser melhor explicitada. Os autores mencionam a questão da desigualdade e influência dos determinantes sociais na mortalidade, sendo o estado da Bahia, um desses exemplos, mas o objetivo do estudo não aponta para esta questão. Ademais, não é apresentado lacunas para justificar o desenvolvimento do estudo, bem como os avanços que poderá trazer.

Métodos:

Oriento justificar o cálculo das taxas por 10000 nascidos vivos, visto que inicialmente dificulta o entendimento, especialmente na figura 1. Isso em função da introdução apontar taxas por 1000 nascidos vivos. 

Recomendo que as características sociodemográficas maternas, obstétricas e do recém-nascido sejam apresentadas com as taxas específicas e não apenas pela proporção.

Resultados:

Atendem o objetivo e métodos propostos.

Discussão:

Aponta para as questões das desigualdades e determinantes sociais da saúde/morte, contudo os dados/resultados poderiam aprofundar estas análises.

pag. 5- linha 53 a 60;

pág. 6- linha 3 a 16- a discussão pode ser alterada ao apresentar os resultados por taxas e não por proporção, conforme recomendado alterar nos métodos.

pág. 7- linha 35- incompletude dos dados- pode ser melhor explorada, não apenas como limitação, mas também como necessidade de melhorar informações básicas faltantes.

pág. 7- linha 38- 39- para superar as limitações do uso de dados agregados, uma opção é o uso da transferência de arquivos no Datasus, tabwin, link seguinte: https://datasus.saude.gov.br/transferencia-de-arquivos/

Em síntese:

O estudo apresenta um relevante atual para a saúde pública, atende ao objetivo proposto, apresenta métodos adequados e atinge os resultados. A discussão é realizada de modo adequado, com outros estudos atuais e com recomendações para a gestão pública.

Apresenta as limitações do estudo.

Contudo, considero que o estudo pode avançar com dados que responda melhor o tema das desigualdades nas taxas de mortalidade neonatal no estado da Bahia, em relação a outros estados e ao Brasil. Pode explorar melhor os dados para resultados que apontam para as determinações sociais da saúde, bem como iniquidades regionais (isso é possível fazer com os dados agregados).

Recomendo estes avanços no estudo.

